# Parameter Optimization of Support Vector Machine to Improve the Predictive Performance for Determination of Aflatoxin B_1_ in Peanuts by Olfactory Visualization Technique

**DOI:** 10.3390/molecules27196730

**Published:** 2022-10-09

**Authors:** Chengyun Zhu, Jihong Deng, Hui Jiang

**Affiliations:** 1 School of Physics and Electronic Engineering, Yancheng Teachers University, Yancheng 224007, China; 2 Jiangsu Intelligent Optoelectronic Devices and Measurement and Control Engineering Research Center, Yancheng 224007, China; 3 School of Electrical and Information Engineering, Jiangsu University, Zhenjiang 212013, China

**Keywords:** peanut, olfactory visualization technique, aflatoxin B_1_, feature optimization, determination

## Abstract

This study proposes a novel method for detection of aflatoxin B_1_ (AFB_1_) in peanuts using olfactory visualization technique. First, 12 kinds of chemical dyes were selected to prepare a colorimetric sensor to assemble olfactory visualization system, which was used to collect the odor characteristic information of peanut samples. Then, genetic algorithm (GA) with back propagation neural network (BPNN) as the regressor was used to optimize the color component of the preprocessed sensor feature image. Support vector regression (SVR) quantitative analysis model was constructed by using the optimized combination of characteristic color components to achieve determination of the AFB_1_ in peanuts. In this process, the optimization performance of grid search (GS) algorithm and sparrow search algorithm (SSA) on SVR parameter was compared. Compared with GS-SVR model, the model performance of SSA-SVR was better. The results showed that the SSA-SVR model with the combination of seven characteristic color components obtained the best prediction effect. Its correlation coefficients of prediction (R_P_) reached 0.91. The root mean square error of prediction (RMSEP) was 5.7 μg·kg^−1^, and ratio performance deviation (RPD) value was 2.4. The results indicate that it is reliable to use the colorimetric sensor array with strong specificity for the determination of the AFB_1_ in peanuts. In addition, it is necessary to properly optimize the parameters of the prediction model, which can obviously improve the generalization performance of the multivariable model.

## 1. Introduction

Peanuts are an import cash crop and oil crop, which are grown on a large scale in all countries in the world [1]. Peanuts are rich in plentiful nutrients such as fat and protein [2]. Finished peanuts are prone to mold contamination and mildew during the circulation and storage process. Among them, aflatoxin is a highly toxic and carcinogenic compound, which is often detected in moldy peanuts. Aflatoxin B_1_ (AFB_1_) is the most toxic [3]. It is currently the most toxic of known molds and poses a high health threat to humans and animals. Humans who ingest even small amounts of AFB_1_ can be poisoned and cause liver damage [4]. Therefore, AFB_1_ is considered to be one of the most typical carcinogens. The limit of the AFB_1_ in peanut and its products in China is 20 μg·kg^−1^. After peanuts are contaminated by the AFB_1_, the value of peanuts will be greatly reduced, and more importantly, the edible safety of peanuts and their products will be affected. Therefore, it is essential to seek a suitable technique for quantitative determination of the AFB_1_ in peanuts.

The traditional detection methods of the AFB_1_ in food mainly include thin layer chromatography [5,6,7], high performance liquid chromatography (HPLC) [8,9,10], and immunological methods [11,12]. Among them, the reversed-phase HPLC method using a fluorescence detector has become the main method for detecting the AFB_1_ [13]. Although the HPLC method has its unique advantages, the preprocessing of sample detection is more complicated and the detection time is long [14]. Meanwhile, the above-mentioned traditional detection methods are all laboratory physical and chemical analysis methods, which are time-consuming, arduous, and costly. These methods are difficult to meet the needs of on-site analysis and detection of the AFB_1_ in modern grains. Therefore, it is very essential to develop a detection method with high efficiency, high accuracy, and strong pertinence to realize the quantitative determination of the AFB_1_ in peanuts.

Colorimetric sensor technology is a novel nondestructive testing technology [15]. It is a senor application technology based on odor sensing developed by Suslick et al. [16]. The method mainly realizes the attribute analysis of the sample by analyzing the color change in the reaction between the color-sensitive material and the experimental sample. In recent years, colorimetric sensor technology has been widely used [17], such as rice quality analysis [18,19,20], tea quality analysis [21,22,23,24], and black tea fermentation process detection [25]. It has also been successfully applied to the identification of mildew stage of mildew wheat [26]. However, there are few reports on the quantitative determination of mycotoxins in cereals at home and abroad. In addition, the core of colorimetric sensor technology is the preparation of color-sensitive sensor arrays with strong specificity. However, there are relatively few related studies on the preparation of sensors, and there is a lack of reliable theoretical guidance. Most of the existing studies are based on experience or through trials to select a suitable sensor array.

Therefore, in this study, some chemical dyes were selected according to the analysis results to prepare specific sensor array. The characteristics of the sensor image data were mined by using the chemometrics methods, and the nonlinear detection was constructed to realize the quantitative determination of the AFB_1_ content in peanuts by the colorimetric sensor technology.

## 2. Results

### 2.1. Division of the Sample Set

In this study, the sample set was divided into two parts: calibration set and prediction set. First, the GA-BPNN algorithm was used to optimize the color sensor array. Considering the randomness of the results of the optimization algorithm, this study independently ran the GA-BPNN algorithm 50 times. In the process of optimizing variables, the sample set was randomly dived into calibration set and prediction set with a partition ratio of 3:1. The second step is to construct an analytical model of SVR using the optimization variables. In order to make the model have better prediction performance, it is more reasonable to divide the calibration set and the prediction set. The specific division rules are as follows: the AFB_1_ values of all 100 samples were arranged in ascending order, and then a group of four samples was randomly selected in order, one of which was put into the prediction set, and the other three were put into the calibration set. This partition method can ensure that the sample features of the calibration set include the sample features of the prediction set. Table 1 show the division of samples during SVR construction. As can be seen from Table 1, there is no significant difference between the average value and standard deviation of the AFB_1_ of the peanut samples in the calibration set and the prediction set.

### 2.2. Response Results of Colorimetric Sensor Array

**Figure 1:**
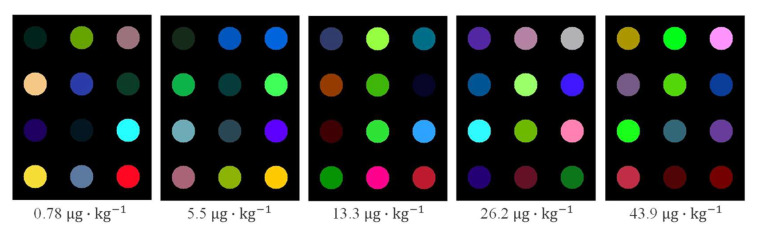
Difference image of peanut colorimetric sensor with different mildew degree.

Figure 1 shows the characteristic image of the colorimetric sensor array of peanut samples with different AFB_1_ content after pretreatment. As can be seen from Figure 1, there are significant differences in sensor feature image of peanut samples with different AFB_1_ contents. Therefore, we can infer that during the moldy process of peanuts, the composition of the volatile substances produced by the peanuts has undergone significant changes, and the content of indicative volatile substance has also undergone specific changes at different stages. These changes are easily detected by senor arrays and are reflected by color differences. It can also be observed from Figure 1 that some color sensitive spots with large color changes can be clearly distinguished by the naked eye. However, with intensification of the mildew process, the color saturation of some points becomes smaller and smaller. This directly indicates that the colorimetric sensor prepared in this study can visualize the changes in volatile substances during peanut mildew. However, some of the color sensitive spots show little difference in the adjacent mildew stage. It may be that some substances release fewer volatile substances or change less during the moldy stage of peanut, resulting in less obvious color reaction. Consequently, it is reliable to achieve determination of the AFB_1_ in peanut using the colorimetric sensor array. The results presented in Figure 1 can also indicate that there may be some information in all color sensitive points. Therefore, before the construction of the SVR detection model, it is necessary to perform further feature processing on the color components of the difference image before and after the reaction of the sensor.

### 2.3. Feature Optimization Results Based on GA-BPNN

**Figure 2:**
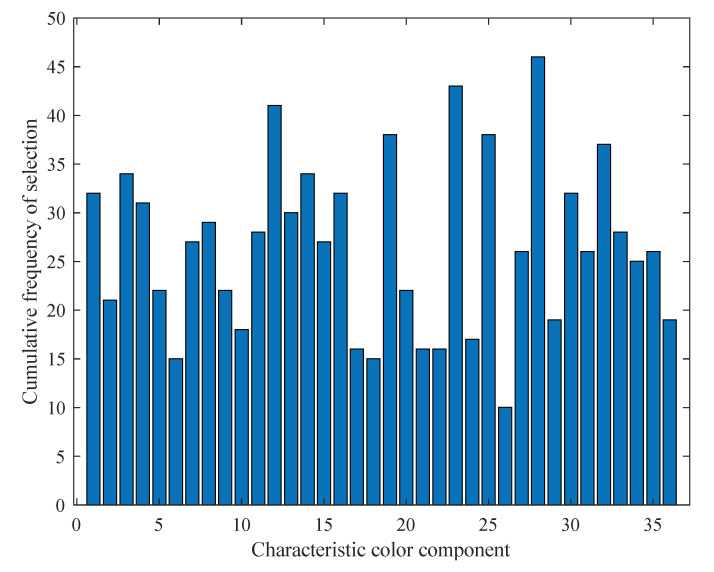
Statistics of the selected times of each color component after the GA-BPNN algorithm was run independently 50 times.

Figure 2 shows that statistics of the selected times of each color component after the GA-BPNN algorithm runs independently for 50 times. The results in Figure 2 show that all feature color components have been selected, and the color components with the lowest frequency have been selected 10 times. The result indirectly shows that the GA retains more variables in each variable optimization process, which may have a certain relationship with the GA optimization criteria. It can be clearly observed from Figure 2 that multiple characteristic color components are selected frequently. Especially for the 12th, 23rd, and 28th color components, their cumulative times all exceed 40. In each variable optimization process, three color components are almost selected, which indicates that three color components may effectively capture the changes in the AFB_1_ in moldy peanuts. They can be used as important color components to construct the AFB_1_ model of moldy peanuts. On the contrary, compared with most color components, some color components have less cumulative frequency during 50 independently runs of the GA-BPNN, such as the 6th, and 26th color components. These color components have relatively little correlation with the content of the AFB_1_ in moldy peanut. Therefore, in the later modeling process, these variables can be eliminated to improve the stability of the model.

From the further analysis in Figure 2, it can be found that there are 13 color components with a frequency of 30 and above, which shows that these color components are closely related to the change in the AFB1 content in moldy peanuts. If these variables are used as the input of the model to rebuild the model, the computational difficulty in the model operation process will be greatly reduced and the prediction accuracy of the model will be further improved. Among them, compared with other color components, there are seven color components more frequently, reaching more than 35 times. They are the 3rd, 12th, 19th, 23rd, 25th, 28th, and 32th color components in order. Among the seven color components, there are three components whose cumulative frequency exceeds 40 times, followed by the 12th, 23rd, and 28th color components. According to the above analysis results, three color components combinations based on 13 color components, 7 color components, and 3 color components are used as model input. Meanwhile, the performance of three models with different input variables is compared.

## 3. Discussion

**Figure 3:**
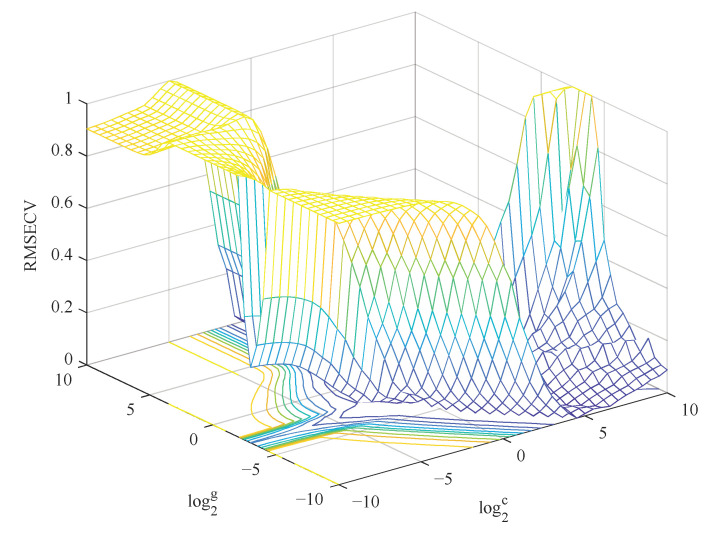
Three-dimensional view of C and g parameter is searched in the SVR using GS method.

Table 2 shows the prediction results of two the SVR models based on different feature combinations. It is clear from the results of the GS-SVR model in Table 2 that the Case 2 prediction model obtains the best prediction performance. Although Case 3 mode selected the least feature color components, the performance of the GS-SVR model established by Case 3 mode was lower than of the GS-SVR model obtained by Case 1 and Case 2 mode. The fact indicates that the intrinsic features of the original data cannot be retained to a certain extent due to too few characteristic variables selected for modeling, which greatly reduces the prediction accuracy of model. Figure 3 shows the process of searching the optimal C and g value optimization of the SVR model based on the RBF kernel function in Case 2 mode. When C = 1.1, g = 0.50, the model achieved the best results, where RMSEP = 5.8, R_P_ = 0.90, and RPD = 2.3.

From the further analysis in Table 2, it can be found that compared with the GS-SVR analysis model, the SSA-SVR has achieved better prediction results overall. The result may be related to the characteristics of the GS and the SSA. The grid search generally consists of two steps. The first step is to set a wide search area in advance and ensure a large stride length. The second step will narrow the step size and search scope on the basis of the first step, so as to better and more accurate optimal solution. The search process can effectively reduce the model running time and calculation difficulty. However, since the objective function is generally non-convex, it is very likely to obtain a local optimum rather than a global optimum. The SSA has high performance search capability, which enables it to search the potential regions of the global optimum, and can effectively avoid the problem of falling into the local optimum. Thus, the model achieves better generalization ability than the GS-SVR model.

**Figure 4:**
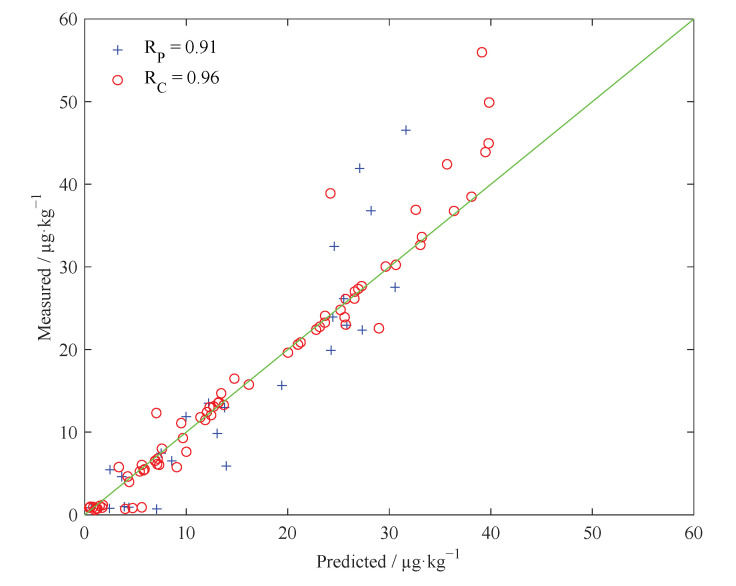
Scatter plot between the predicted value of the AFB_1_ in peanuts by the optimal SSA-SVR model and the reference measured value.

For the SSA-SVR model, the results in Table 2 indicate that the SSA-SVR model obtained in the Case 2 mode performs the best. In addition, the performance of the SSA-SVR model established in Case 3 mode is lower than the performance of the SSA-SVR model established Case 1 and Case 2, which is consistent with the performance result of the GS-SVR model in different modes. It can be seen that although the model structure can be simplified by using fewer feature variables for modeling, the overall prediction accuracy of the SVR model is reduced to a certain extent. Considering the comprehensive performance of the model, we believe that the SSA-SVR model established in Case 2 mode is the best model. Figure 4 shows the scatter plot of the prediction results and actual measurement results of the best SSA-SVR model in the calibration set and prediction set. In the model, R_P_ = 0.91, and RPD = 2.4. In addition, it can also be seen from Figure 4 that the best SSA-SVR model does not have a very good prediction effect on peanut samples with higher AFB_1_ concentrations. We believe that this may be related to the actual measurement error caused by human factors during the physical and chemical determination of AFB_1_ in peanuts. Therefore, accurate experimental data are the premise to ensure good model calibration. However, this does not affect the purpose of our current study.

Furthermore, there are some studies on the application of olfactory sensors to realize the quality monitoring of mildew grain in the existing literature. For example, Gobbi et al. used electronic nose to detect high fumonisin content and to predict fumonisin concentration in maize. The electronic nose could correctly recognize high and low fumonisin content of maize cultures and provide a fair quantitative estimation [27]. Paolesse et al. studied the possibility of the application of electronic nose for an early detection of volatile compounds in infected samples and to discriminate between non-infected and infected samples with two different species of fungi (*Penicillium chrysogenum* and *Fusarium verticillioides*). The results showed that the electronic nose has certain ability to track the changes in headspace caused by fungal contamination [28]. Leggieri et al. evaluated the potential use of an electronic nose for rapid identification of mycotoxin contamination above legal limits in maize samples. The results confirmed the application of e-nose, combined with ANN, as a reliable assessment for AF and FB contamination in maize [29]. The above studies proved the potential of olfactory sensor technology in grain mildew quality monitoring. However, most of these studies are still in the level of qualitative analysis or semi-quantitative analysis, and do not realize quantitative analysis in a real sense. In our current study, we achieved the quantitative determination of AFB_1_ in peanut using olfactory visualization technology. Although the model results need to be improved, they directly demonstrate the potential of olfactory sensor technology for quantitative analysis of mycotoxin contamination levels in cereals.

## 4. Materials and Methods

### 4.1. Moldy Peanut Sample Preparation

Fresh peanuts (Baisha, Liaoning, China) were purchased from a large local market, a total of 10 kg. The purchased peanuts were spread flat on two metal trays. The two trays were then placed in a constant temperature and humidity equipment (HWS-250W, Tianjin Hongnuo Instrument Co., Ltd., Tianjin, China). Here, the temperature and relative humidity of the HWS-250W were set to 28 °C and 80%, respectively. Daily appropriate samples were taken for physical and chemical experiments to determine the content of the AFB_1_. Starting from the determination of the AFB_1_ in peanuts (day 5), 20 peanut samples were taken from different position on the two trays every other day, and each sample was 10 g. After the 8th day, the degree of mildew in the peanut sample was visible to the naked eye, and the sampling was terminated. In this way, a total of 100 peanut samples with different AFB_1_ contents were collected in the experiment.

### 4.2. Determination of the Aflatoxin B_1_

For each peanut sample collected, the peanut sample was tested for the AFB_1_ in accordance with the second method in GB 5009.22-2016.

### 4.3. Colorimetric Sensor Array Preparation

Based on the team’s previous research experience, some appropriate chemical dyes were selected to prepare a colorimetric sensor array to capture the changing information of moldy peanuts. The names of the porphyrin materials used to prepare the sensor arrays are listed in Table S1.

The substrate prepared by the colorimetric sensor array is polyvinylidene fluoride (PVDF) membrane (Millipore, USA). The specific manufacturing process is as follow: (1) PVDF was cut into a rectangular base material of 4 cm × 3 cm for standby. (2) Dichloromethane was used as the solvent, and 12 kinds of porphyrin materials were dissolved in it respectively to obtain 2 mg/mL solution. After 10 min of ultrasound, the solution was kept in a cool dark place for reserve. (3) One microliter of solution was extracted from 100 mm×0.3 mm capillaries and dotted on hydrophobic PVDF with array template assist. After standing for a period of time, the colorimetric sensor array can be obtained.

### 4.4. Sensor Data Collection and Preprocessing

Figure S1 shows the data acquisition and data preprocessing process of the olfactory visualization system. First, a scanner was used to acquire the image before the colorimetric sensor reacts with the sample. Then, a 10 g peanut power crushed by a multifunctional crusher (BJ-150, Deqing Baijie Electric Appliance Co., Ltd., Deqing, China) was put into the prepared petri dish. Next, the peanut sample and colorimetric sensor were sealed in a petri dish using plastic wrap. After the sample reacted with the sensor for 16 min, the sensor array was removed. Finally, the image information after the reaction between the colorimetric sensor and the sample was obtained by using the flat plate scanner.

MATLAB software (Matlab R2016a, MathWorks, Natick, MA, USA) was used to filter the image information of colorimetric sensor array before and after the reaction. Then, the gray mean values of red (R), green (G), and blue (B) components within the radius of 12 pixels around the colored sensitive points were extracted, respectively, and normalized to 0–255. Then, the gray difference before and after the change in all kinds of sensitive points is calculated. In this way, three color difference components ΔR, ΔG, and ΔB of each corresponding color sensitive point can be obtained. Finally, the obtained ΔR, ΔG and ΔB were normalized, and the gray-scale image was superimposed to generate the characteristic image of the colorimetric sensor. In this study, each colorimetric sensor array had 12 color sensitive points, and each color sensitive point can obtain 3 color feature components. Thus, the colorimetric sensor eigenvalues of each peanut sample had 36 color components.

### 4.5. Data Analyses Methods

#### 4.5.1. Back Propagation Neural Network

Back propagation neural network (BPNN) is one of the most classic neural network algorithms, and it is a neural network training system for calculating backpropagation errors [30]. The main feature of the BPNN network is that the signal is forwarded, and the error is propagated back. The weights of the network are optimized through iteration to make the BPNN predicted output consistent with the expected output as much as possible. In the process of continuous updating and innovation, BPNN algorithm has gradually formed a huge learning system, which can deal with complex system. It has three main parts, namely input layer, hidden layer, and output layer. BPNN has a forward pass process and a back pass process. In the forward pass stage, the signal source is transmitted layer by layer in turn and finally reaches the output layer. The input signal is processed at each layer to finally generate the output signal. The result obtained is compared with the expected output, an error is measured, and then the process of back propagation begins. In the forward transmission of signals, neurons in each layer are activated and affect the state of neurons in the next layer. Network weights are not updated during process. In the back-propagation stage, the output error results will be transmitted back to the input layer by layer, thereby affecting the weights of each node. BPNN continuously revises the network weights in the learning process, which is repeated until the preset learning times and learning errors are reached.

In this study, BPNN needs to initialize some parameter before running. For the BPNN, the number of hidden layer neurons: 10; the learning rate: 0.10; the momentum factor: 0.95; the initial weight: 0.30; the minimum root mean square error: 0.0010; and the maximum number of training times: 100.

#### 4.5.2. Genetic Algorithm

Genetic algorithm (GA) is an intelligent optimization algorithm based on the survival of the fittest genetic mechanism in nature [31]. The characteristic of genetic algorithm is that it operates directly with no objects. Unlike some algorithms, it has no constrains on derivation and continuity of function. During the whole running process, it can adjust the search direction spontaneously, and there is no definite search direction. These properties make the algorithm have better global optimization ability. In the GA algorithm, population and individual are important concepts. A population represents a collection of solution to a problem. Individuals form a population through some genetic coding. Each individual undergoes genetic manipulations such as replicative mutation. The operation process of GA algorithm is as follows: (1) Firstly, the maximum number of iterations and the initial count of evolutionary iterators are set, the number of populations are randomly generated as the initial population, and the fitness of each individual in the population is calculated. (2) The selection operation is performed on the population, and the optimized individuals are inherited to the next generation through the new individuals generated by the pairing and crossover, and the quality of the optimized population is continuously updated and iteratively updated. Cross operation is performed on the species population to generate new offspring. (3) The next generation group is obtained by updating the group with mutation operation. (4) Repeat the above operations. When the counter number reaches the maximum number of iterations, the individual with the greatest fitness obtained in the evolution process is used as the optimal solution output, and the calculation is terminated.

In this study, The GA is used for feature optimization in the first step of colorimetric sensor model construction. Taking into account the randomness of the GA, the GA algorithm is run 50 times to eliminate the effect of randomness on the optimization results. Here, the GA parameters are set as follows: the population size is set to 20, the crossover probability and the mutation probability are set to 0.70 and 0.10, respectively, and the maximum number of iterations is set to 100.

#### 4.5.3. Support Vector Regression

Support vector regression (SVR) is a promotion of support vector machine (SVM), which is specially used to deal with regression analysis problems [32]. For nonlinear regression problems, SVR mainly maps the input sample space to a high-dimensional feature space by introducing an appropriate kernel function, and then constructs a regression estimation function in this new space. In other words, it is trying to find an optimal classification hyperplane so that the error of all training samples from the optimal hyperplane is minimized. The SVR algorithm reduces the estimation problem to a convex quadratic programming problem with linear equality constraints and linear inequality constraints, which can ensure the global optimality of the algorithm. The SVR algorithm is based on modern statistics, mainly for small samples, and its optimal solution is based on limited sample information. From this point of view, the use of SVR to build the model in this study is very suitable.

In this study, radial basis function (RBF) is used as the kernel function, and the penalty coefficient C and RBF kernel function parameter g of the SVR are optimized by using the five-fold cross-validation combined with grid search.

#### 4.5.4. Parameter Optimization Algorithm

Grid search (GS) is the simplest and most widely used hyperparameter search algorithm [33]. The method optimizes the estimated function parameters through a cross-validation method to achieve the optimal learning algorithm.

**Figure 5:**
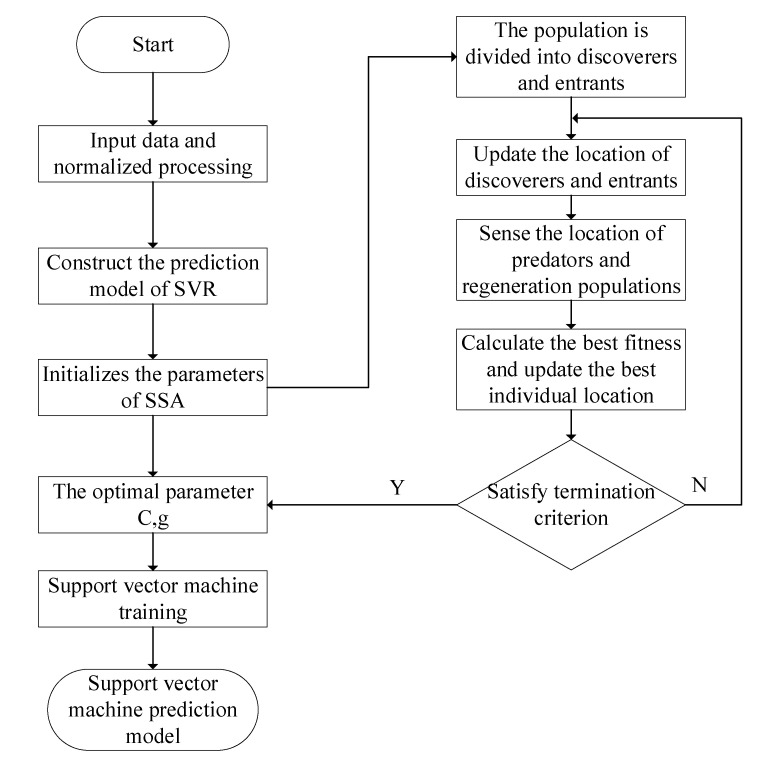
The specific flow chart of the SVR prediction model based on SSA.

Sparrow search algorithm (SSA) is an intelligent optimization algorithm proposed in 2020 [34]. Sparrows are omnivorous gregarious birds. Compared with other birds, sparrows are relatively intelligent and have better memory. In daily life, sparrows are divided into producers and beggars. The producers actively seek food, while the beggars obtain food from the producers. Through this strategy of producers and beggars, sparrows are able to obtain food to survive. Inspired by the foraging behavior of sparrows, the specific optimization process of the algorithm is shown in Figure 5.

In this study, C and g in the SVR model are mainly optimized., and the model results are compared. Here, when the GS optimizes the parameters C and g of the SVR, their value ranges are 2^−10^,2^−9.5^, …, 2^9.5^, 2^10^. When the SSA optimizes the parameters C and g of the SVR, the population size is 20 and the maximum number of iterations is 100. The value range of C is set to 0.001–100, and the value range of g is set to 0.0010–1000.

#### 4.5.5. Model Evaluation

In this study, in order to evaluate the performance of the established multiple regression model, correlation coefficient of calibration (R_C_), correlation coefficient of prediction (R_P_), root mean square error of calibration (RMSEC), and root mean square error of prediction (RMSEP) are mainly used. In addition, in order to standardize the prediction accuracy, the ratio performance deviation (RPD) of the SSA-SVR model is calculated. When the RPD value is less than 1.4, the prediction performance of the model is considered to be poor. Their specific calculation formula is as follows:

(1) $$\mathrm{RMSEC}=\sqrt{\frac{\sum_{i=1}^{N_{cal}}{\left(y_{i}-\tilde{y_{i}}\right)}^{2}}{N_{cal}}}$$

(2) $$R_{C}=\sqrt{1-\frac{\sum_{i=1}^{N_{cal}}{\left(y_{i}-\tilde{y_{i}}\right)}^{2}}{\sum_{i=1}^{N_{cal}}{\left(y_{i}-\bar{y_{i}}\right)}^{2}}}$$

(3) $$\mathrm{RPD}=\frac{\mathrm{SD}}{\mathrm{RMSEP}}$$

In the above formula, $y_{i}$, $\tilde{y_{i}}$ and $\bar{y_{i}}$ represent the measured AFB_1_ value, the predicted AFB_1_ value and the average AFB_1_ value in the calibration set, respectively. $N_{cal}$ is the number of samples of the corresponding data set. Similarly, the values of RMSEP and R_P_ can be calculated. In the third formula, SD is the standard deviation of the AFB_1_ values of all peanut samples in the prediction set.

## 5. Conclusions

In this study, a special colorimetric sensor array was prepared for the indicative characteristic volatile substances in the process of peanut mold, which was used for determination of AFB_1_ in peanut. The GA-BPNN was employed to select the feature combinations of the sensor, the optimal characteristic color components are obtained; the SVR model of optimal combination of different color components is established, and the effects of the GS and the SSA optimization algorithm on the SVR parameter optimization are compared combine. The results of the study show that compared with the GS-SVR model, the SSA-SVR has better prediction performance. The SSA-SVR model with seven characteristic color components obtains the best prediction results. Its R_P_ reaches 0.91, and the RPD value is 2.4. This study has shown that it is reliable to achieve the quantitative determination of the AFB_1_ in peanut by colorimetric sensor technology combined with appropriate stoichiometry method. This research can provide another technical method for quantitative and on-site detection of mycotoxins in peanut and other cereals during storage.

## Figures and Tables

**Table 1:** The AFB_1_ values measurement result in the calibration and prediction sets.

Subsets	Sample Number	Units	Minimum	Maximum	Mean	Standard Deviation
Calibration set	75	$\mu g\;{\mathrm{kg}}^{-1}$	0.60	56.0	16.2	13.8
Prediction set	25	$\mu g\;{\mathrm{kg}}^{-1}$	0.71	46.5	15.9	13.6

**Table 2:** Results of the different SVR models based on different combinations of color components.

Model	Mode	Number of Variables	Parameter Combination	Calibration Set		Validation Set		
				R_C_	RMSEC	R_P_	RMSEP	RPD
GS-SVR	Case 1	13	C = 0.50 g = 0.18	0.91	5.7	0.89	6.1	2.2
	Case 2	7	C = 1.1 g = 0.50	0.94	4.5	0.90	5.8	2.3
	Case 3	3	C = 5.7 g = 0.35	0.81	8.0	0.81	8.0	1.7
SSA-SVR	Case 1	13	C = 17.9 g = 1.5	0.94	4.7	0.91	5.8	2.3
	Case 2	7	C = 50.3 g = 1.56	0.96	3.4	0.91	5.7	2.4
	Case 3	3	C = 85.8 g = 11.6	0.86	7.1	0.75	9.2	1.5

## Data Availability

The data is currently classified and will be available in 2024 with permission from the project.

## Supplementary Materials

The following supporting information can be downloaded at: https://www.mdpi.com/article/10.3390/molecules27196730/s1, Table S1: The names of porphyrin materials used to prepare colorimetric sensor arrays. Figure S1: Data acquisition and pretreatment process of the olfactory visualization system.

## Sample Availability

Available for request.
