# Supplementary material for: Parameter Optimization of Support Vector Machine to Improve the Predictive Performance for Determination of Aflatoxin B1 in Peanuts by Olfactory Visualization Technique
Source: Molecules. 2022 Oct 9;27(19):6730. doi: 10.3390/molecules27196730 (PMC9573054; doi:10.3390/molecules27196730)
Supplement: Supplementary file 1 [file molecules-27-06730-s001.zip › molecules-1931213-supplementary.pdf]

Table S1 The names of porphyrin materials used to prepare colorimetric sensor arrays.

Number	Name
1	5,10,15,20-Tetraphenyl-21H,23H-porphine
2	5,10,15,20-Tetrakis(4-methoxyphenyl)-21H,23H-porphine iron (III) chloride
3	5,10,15,20-Tetraphenyl-21H,23H-porphine iron (III) chloride
4	5,10,15,20-Tetraphenyl-21H,23H-porphine copper (II)
5	5,10,15,20-Tetrakis(4-methoxyhenyl)-21H,23H-porphine cobalt (II)
6	5,10,15,20-Tetraphenyl-21H,23H-porphine zinc
7	meso- Tetraphenyl porphyrin (chlorin free)
8	2,3,7,8,12,13,17,18-Octaethyl-21H,23H-porphine copper (II)
9	2,3,7,8,12,13,17,18-Octaethyl-21H,23H-porphine
10	5,10,15,20-Tetraphenyl-21H,23H-porphine vanadium (IV) oxide
11	5,10,15,20- Tetraphenyl -21H,23H-porphine ruthenium (II) carbonyl
12	2,3,7,8,12,13,17,18-Octaethyl-21H,23H-porphine ruthenium (II) carbonyl

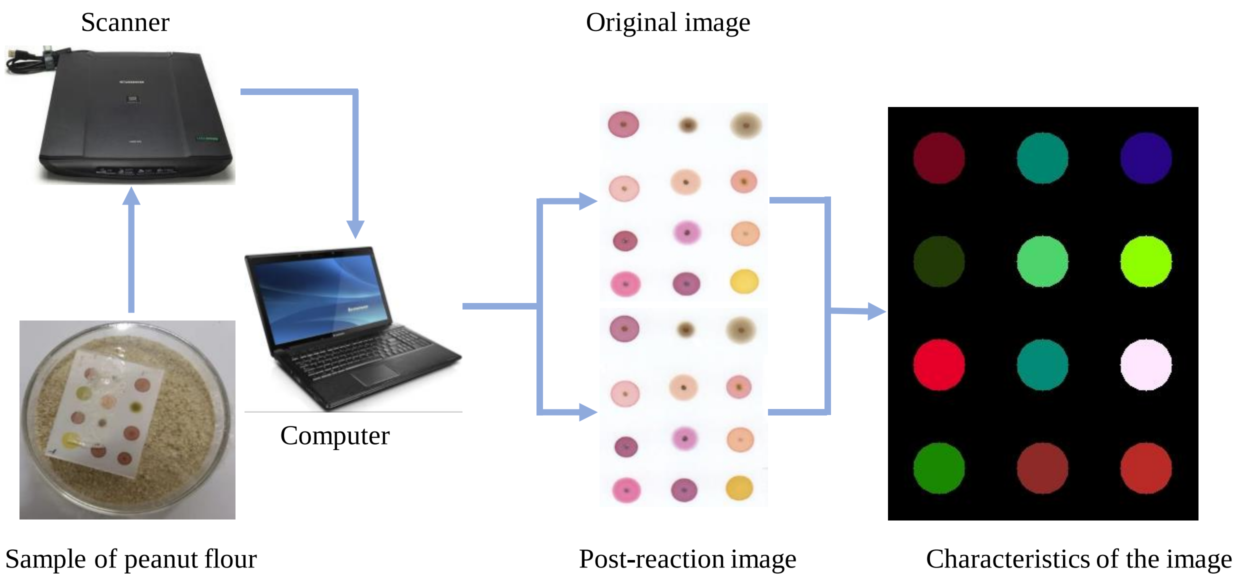

Figure S1 Data acquisition and pretreatment process of the olfactory visualization system.
